# Erratum: Upregulated Fcrl5 disrupts B cell anergy and causes autoimmune disease

**DOI:** 10.3389/fimmu.2023.1356364

**Published:** 2024-01-08

**Authors:** Frontiers Production Office

**Affiliations:** Frontiers Media SA, Lausanne, Switzerland

**Keywords:** Fc receptor-like 5 (Fcrl5), B cells, age/autoimmunity-associated B cells, autoimmunity, immune tolerance, anergy

Due to a production error, the word ‘and’ was left out of the title. The correct title is: ‘Upregulated Fcrl5 disrupts B cell anergy and causes autoimmune disease’.

The publisher apologizes for this mistake.

The original version of this article has been updated.

