# Supplementary material for: Upregulated Fcrl5 disrupts B cell anergy and causes autoimmune disease
Source: Front Immunol. 2023 Sep 28;14:1276014. doi: 10.3389/fimmu.2023.1276014 (PMC10569490; doi:10.3389/fimmu.2023.1276014)
Supplement: Supplementary file 1 [file DataSheet_1.pdf]

Supplementary Material

Supplementary Figures

Supplementary Figure 1

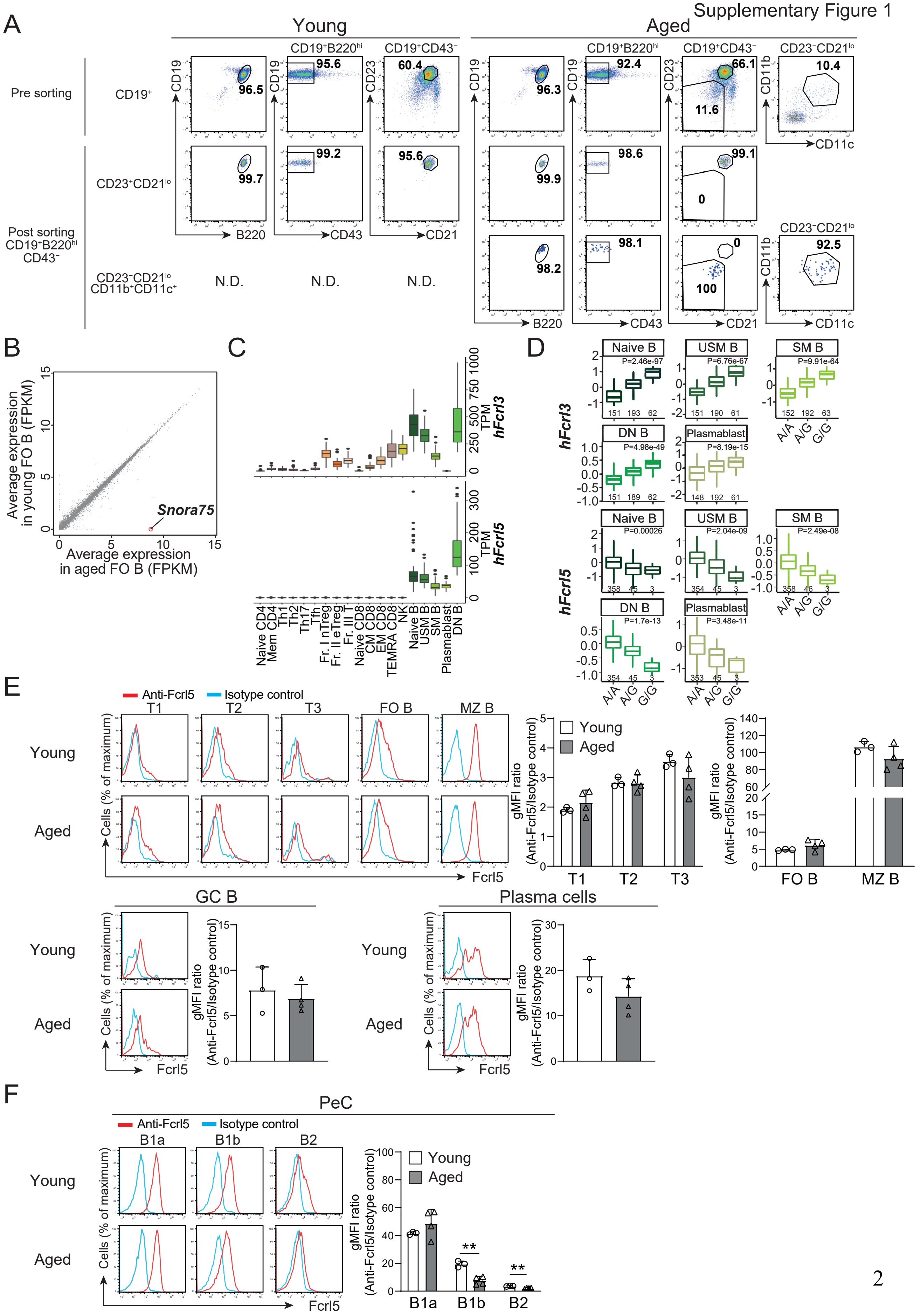

Supplementary Figure 2

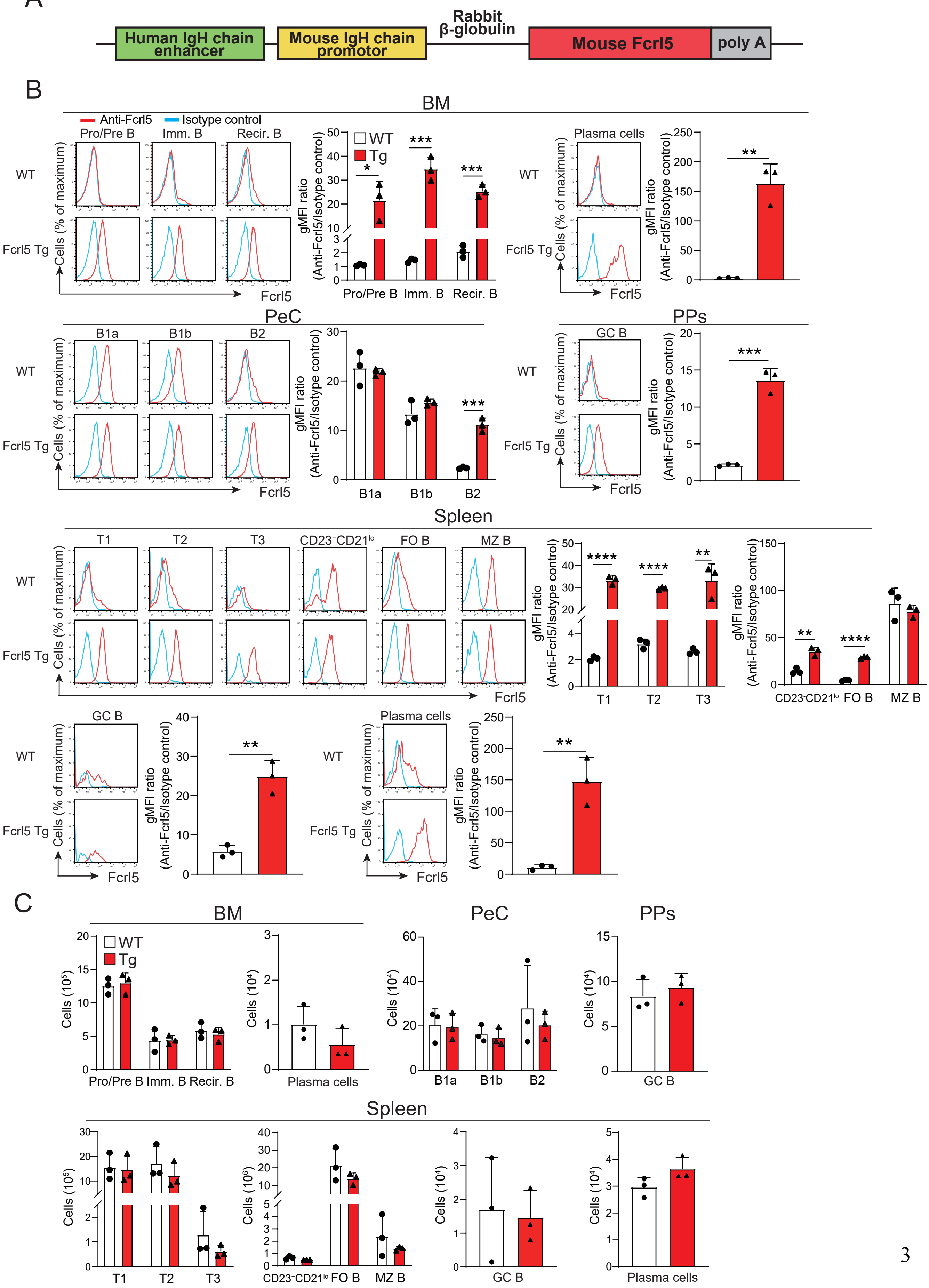

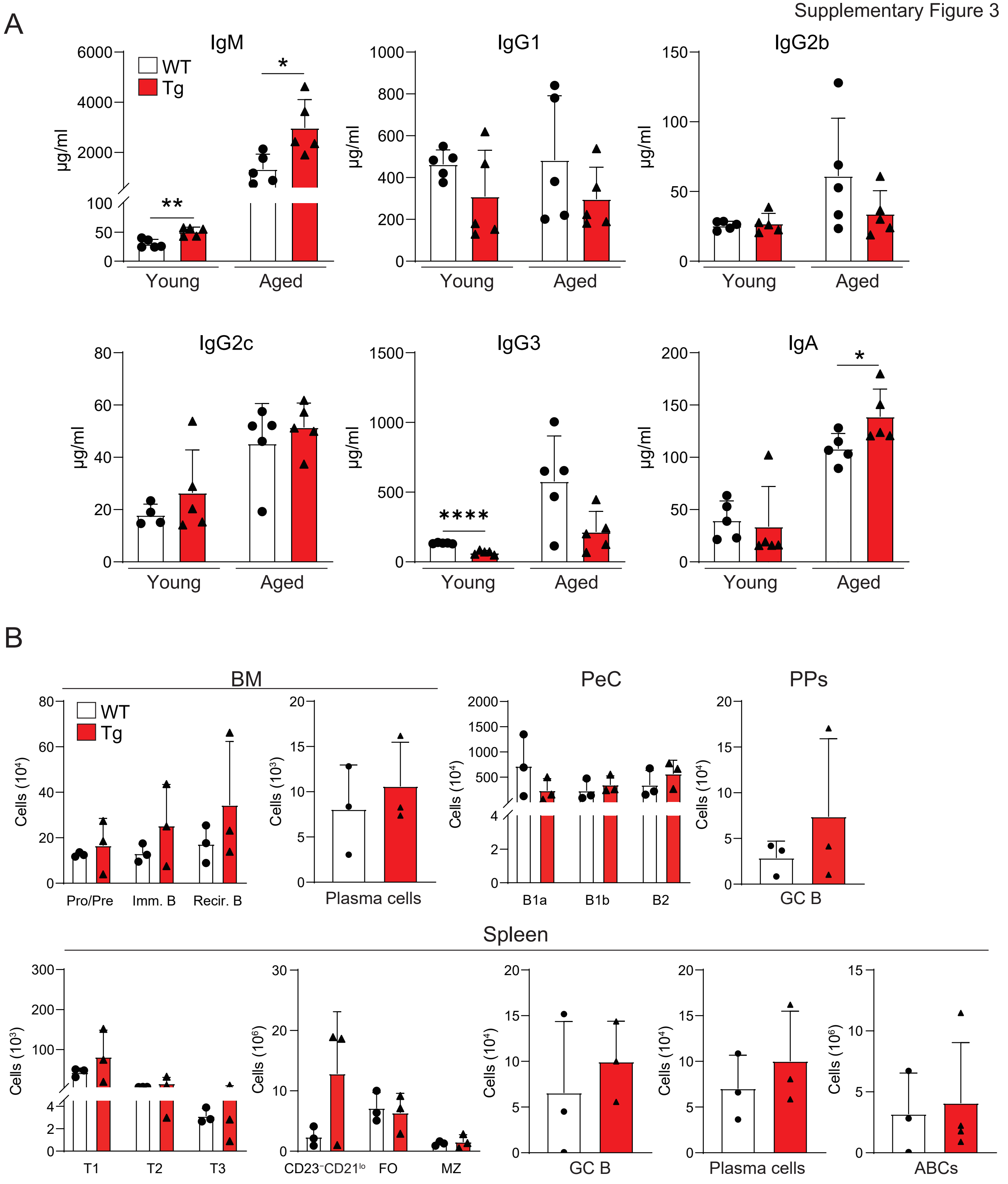
A
IgM
WT
Tg
μg/ml
*
**
Young
Aged
IgG1
IgG2b
IgG2c
IgG3
****
IgA
B
BM
Cells (10⁴)
Pro/Pre
Imm. B
Recir. B
Cells (10³)
Plasma cells
PeC
B1a
B1b
B2
PPs
GC B
Spleen
T1
T2
T3
Cells (10⁶)
CD23⁻CD21ˡᵒ
FO
MZ
GC B
Plasma cells
ABCs

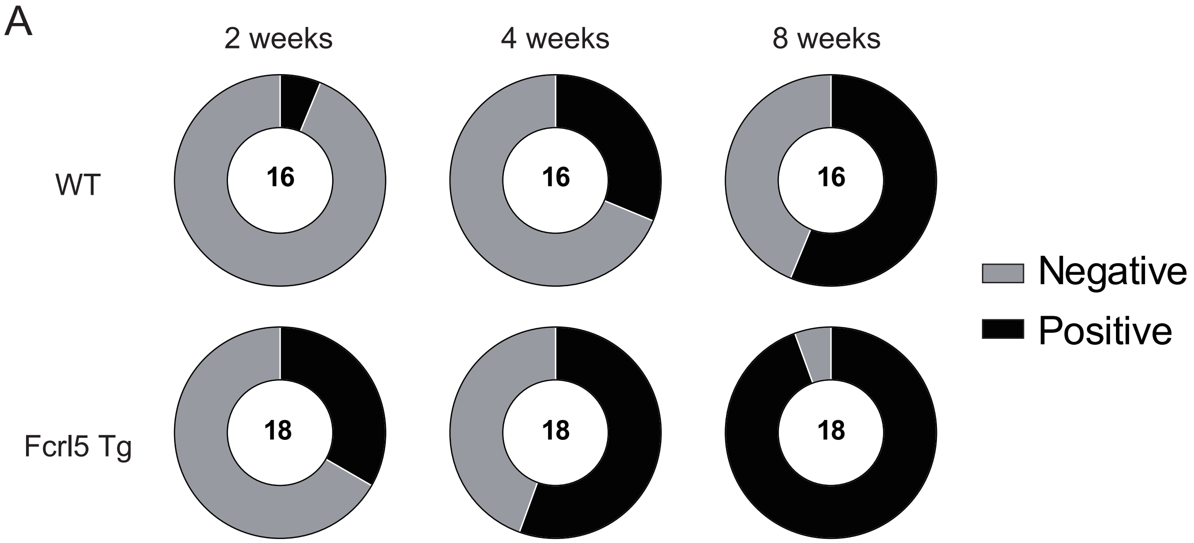
A
2 weeks
4 weeks
8 weeks
WT
16
16
16
Fcrl5 Tg
18
18
18
Negative
Positive

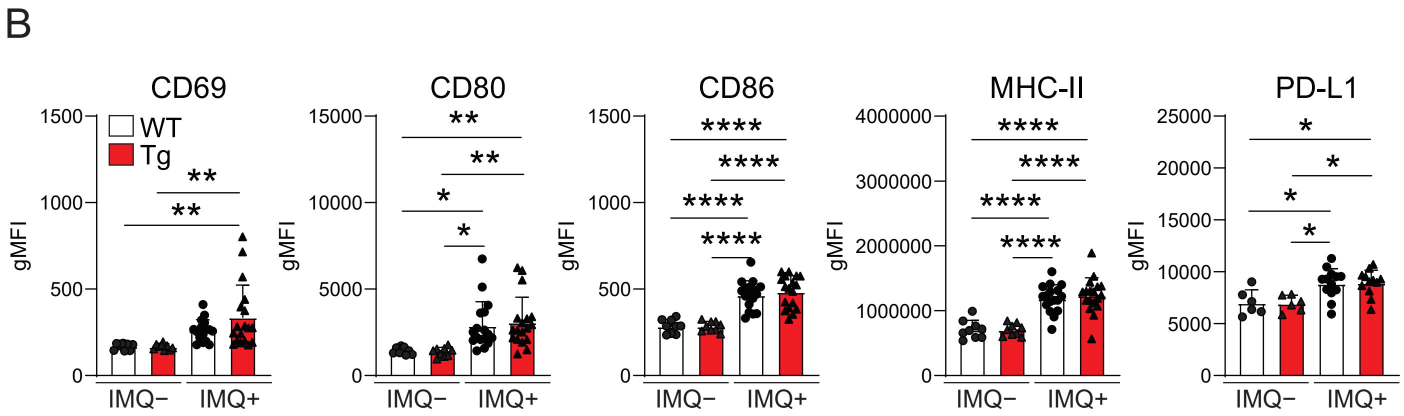
B
CD69
CD80
CD86
MHC-II
PD-L1
WT
Tg
gMFI
IMQ−
IMQ+

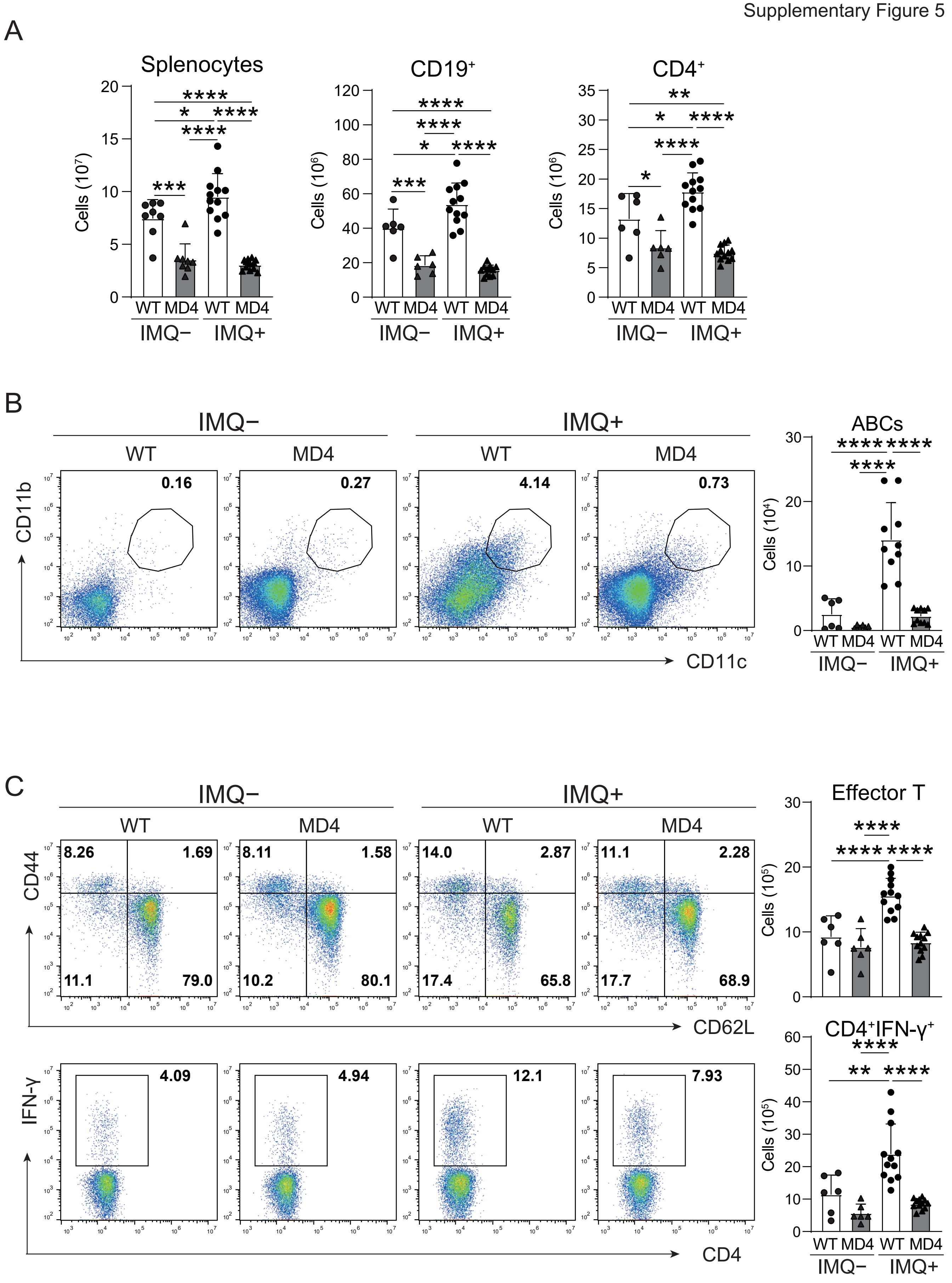
A
Splenocytes
CD19+
CD4+
Cells (10^7)
Cells (10^6)
WT MD4 WT MD4
IMQ− IMQ+
B
IMQ−
IMQ+
WT
MD4
CD11b
CD11c
0.16
0.27
4.14
0.73
ABCs
Cells (10^4)
C
CD44
CD62L
8.26 1.69 11.1 79.0
8.11 1.58 10.2 80.1
14.0 2.87 17.4 65.8
11.1 2.28 17.7 68.9
Effector T
Cells (10^5)
IFN-γ
CD4
4.09
4.94
12.1
7.93
CD4+IFN-γ+

Supplementary Figure legends

Supplementary Figure 1. Gating strategy and distribution of Fcrl5 expression. (A) Gating strategy employed to sort FO B ($CD19^+B220^{hi}CD43^-CD23^+CD21^{lo}$) cells and ABCs ($CD19^+B220^{hi}CD43^-CD23^-CD21^{lo}CD11b^+CD11c^+$) from young and aged WT mice (n=3 mice each). (B) Differential gene expression of FO B cells from young WT mice and FO B cells from aged WT mice (n=3 mice each) presented as a scatter plot. (C) Expression of *hFcrl3* and *hFcrl5* in immune subsets: unswitched memory B (USM B), switched memory B (SM B), and double negative B (DN B) cells in healthy individuals (n=79). TPM, Transcript per million. (D) eQTL effect of GWAS SNP rs7528684 (*hFcrl3*) and rs11264750 (*hFcrl5*) in the immune subsets from patients with 10 immunological disorders (n=416). P-values were obtained by testing the alternative hypothesis that the slope of a linear regression model between genotype and expression deviates from 0. (E) Representative histograms and gMFIs of Fcrl5 expression on T1 ($CD19^+CD93^+IgM^+CD23^-$), T2 ($CD19^+CD93^+IgM^+CD23^+$), T3 ($CD19^+CD93^+IgM^-CD23^+$), FO B ($CD19^+CD93^-CD23^+CD21^{lo}$), MZ B ($CD19^+CD93^-CD23^-CD21^{hi}$), GC B ($B220^+Fas^+GL7^+$), and plasma ($CD138^+TACI^+$) cells from young WT (n=3) and aged WT mice (n=4). Data are representative of two independent experiments. (F) Representative histograms and gMFIs of Fcrl5 expression of B1a ($CD19^+CD5^+CD23^-$), B1b ($CD19^+CD5^-CD23^-$), and B2 ($CD19^+CD5^-CD23^+$) cells in PeC from young WT (n=3) and aged WT (n=4) mice. Data are representative of two independent experiments. Statistical data are shown as mean values with s.d., and data were analyzed by unpaired Student's t-test. **$P<0.01$.

Supplementary Figure 2. Analysis of Fcrl5 expression and B cell development in young Fcrl5 Tg mice. (A) A scheme of the constructs used to generate Fcrl5 Tg mice. (B) Representative histograms and gMFIs of Fcrl5 expression on Pro/Pre B ($B220^+IgM^-$), Immature B (Imm. B; $B220^{lo}IgM^+$), recirculating B (Recir. B; $B220^{hi}IgM^+$) and plasma cells ($CD138^+TACI^+$) in BM, B1a ($CD19^+CD5^+CD23^-$), B1b ($CD19^+CD5^-CD23^-$), and B2 ($CD19^+CD5^-CD23^+$) cells in PeC, GC B ($B220^+Fas^+GL7^+$) in PPs and T1 ($CD19^+CD93^+IgM^+CD23^-$), T2 ($CD19^+CD93^+IgM^+CD23^+$), T3 ($CD19^+CD93^+IgM^-CD23^+$), $CD23^-CD21^{lo}$ ($CD19^+CD93^-CD23^-CD21^{lo}$), FO B ($CD19^+CD93^-CD23^+CD21^{lo}$), MZ B ($CD19^+CD93^-CD23^-CD21^{hi}$), GC B ($B220^+Fas^+GL7^+$), and plasma ($CD138^+TACI^+$) cells in spleen from young WT and young Fcrl5 Tg mice (n=3 mice each). Data are representative of four independent experiments. (C) Absolute numbers of B cell subsets described in (B) from young WT and young Fcrl5 Tg mice (n=3 mice each). Data are representative of five independent experiments. Statistical data are shown as mean values with s.d., and data were analyzed by unpaired Student's t-test. *$P<0.05$, **$P<0.01$, ***$P<0.001$, ****$P<0.0001$.

Supplementary Figure 3. Analysis of natural antibody titers in young and aged Fcrl5 Tg mice and B cell development in aged Fcrl5 Tg mice. (A) Antibody production in serum isolated from young WT, young Fcrl5 Tg, aged WT, and aged Fcrl5 Tg mice (n=5 mice each), assayed by ELISA. Data are pooled from two independent experiments. (B) Absolute numbers of Pro/Pre B ($B220^+IgM^-$), Immature B (Imm. B; $B220^{lo}IgM^+$), recirculating B (Recir. B; $B220^{hi}IgM^+$), and plasma cells ($CD138^+TACI^+$) in BM, B1a ($CD19^+CD5^+CD23^-$), B1b ($CD19^+CD5^-CD23^-$), and B2 ($CD19^+CD5^-CD23^+$) cells in PeC, GC B ($B220^+Fas^+GL7^+$) in PPs and T1 ($CD19^+CD93^+IgM^+CD23^-$), T2 ($CD19^+CD93^+IgM^+CD23^+$), T3 ($CD19^+CD93^+IgM^-CD23^+$), $CD23^-CD21^{lo}$ ($CD19^+CD93^-CD23^-CD21^{lo}$), FO B ($CD19^+CD93^-CD23^+CD21^{lo}$), MZ B ($CD19^+CD93^-CD23^-CD21^{hi}$), GC B ($B220^+Fas^+GL7^+$), plasma ($CD138^+TACI^+$) cells, and ABCs ($CD19^+B220^{hi}CD23^-CD21^{lo}CD11b^+CD11c^+$) in spleen from aged WT (n=3) and aged Fcrl5 Tg (n=3–4) mice. Data are representative of two or three independent experiments. Statistical data are

shown as mean values with s.d., and data were analyzed by Mann-Whitney test. *P<0.05, **P<0.01, ****P<0.0001.

Supplementary Figure 4. Autoantibody production and B cell activation in imiquimod-treated Fcrl5 Tg mice. (A) ANAs production in serum from imiquimod-treated (IMQ+) WT (n=16) and Fcrl5 Tg (n=18) for 2, 4, and 8 weeks, detected by immunofluorescence assay using HEp-2 cells. Data are pooled from three independent experiments. (B) Representative histograms and gMFIs of CD69, CD80, CD86, MHC-II, and PD-L1 expression levels on splenic $CD19^+$ cells isolated from imiquimod-untreated (IMQ–) WT (n=9) and Fcrl5 Tg (n=9) or -treated (IMQ+) WT (n=17) and Fcrl5 Tg (n=18) mice. Data are pooled from three independent experiments. Statistical data are shown as mean values with s.d., and data were analyzed by one-way ANOVA with Tukey's multiple comparisons test. *P<0.05, **P<0.01, ****P<0.0001.

Supplementary Figure 5. Requirement of antigen recognition via BCR for imiquimod-induced inflammation. (A) Absolute number of splenocytes, $CD19^+$, and $CD4^+$ cells in spleen from imiquimod-untreated (IMQ–) WT (n=6–8) and MD4 Tg (n=6–8) or -treated (IMQ+) WT (n=12) and MD4 Tg (n=12) mice. Data are pooled from two independent experiments. (B) Representative flow cytometry plots and absolute numbers of ABCs ($CD19^+B220^{hi}CD23^-CD21^{lo}CD11b^+CD11c^+$) from imiquimod-untreated (IMQ–) WT (n=6) and MD4 Tg (n=6) or -treated (IMQ+) WT (n=10) and MD4 Tg (n=10) mice. Data are pooled from two independent experiments. (C) Representative flow cytometry plots (left) and absolute numbers (right) of $CD4^+$ T cell subsets: effector T ($CD4^+CD25^-CD62L^-CD44^{hi}$) and $CD4^+IFN\text{-}\gamma^+$ cells from imiquimod-untreated (IMQ–) WT (n=6) and MD4 Tg (n=6) or -treated (IMQ+) WT (n=12) and MD4 Tg (n=12) mice. Data are pooled from two independent experiments. Statistical data are shown as mean values with s.d., and data were analyzed by one-way ANOVA with Tukey's multiple comparisons test. *P<0.05, **P<0.01, ***P<0.001, ****P<0.0001.
